# Anti Phospholipase A2 Receptor 1 Antibodies and Membranous Nephropathy Recurrence After Kidney Transplantation

**DOI:** 10.1016/j.ekir.2024.09.012

**Published:** 2024-09-23

**Authors:** Marion Cremoni, Maxime Teisseyre, Olivier Thaunat, Céline Fernandez, Christine Payre, Alan Moutou, Hadi Zarif, Vesna Brglez, Laetitia Albano, Valérie Moal, Georges Mourad, Emmanuel Morelon, Bruno Hurault de Ligny, Philippe Zaoui, Eric Rondeau, Nacera Ouali, Pierre Ronco, Bruno Moulin, Laura Braun-Parvez, Antoine Durrbach, Anne-Elisabeth Heng, Philippe Grimbert, Didier Ducloux, Gilles Blancho, Pierre Merville, Gabriel Choukroun, Yannick Le Meur, Cécile Vigneau, Christophe Mariat, Lionel Rostaing, Jean-François Subra, Jean-Luc Taupin, Gérard Lambeau, Vincent Esnault, Antoine Sicard, Barbara Seitz-Polski

**Affiliations:** 1 Centre de Référence Maladies Rares Syndrome Néphrotique Idiopathique, Centre Hospitier Universitaire de Nice, Nice, France; 2 Unité de Recherche Clinique Côte d'Azur (UR2CA), Université Côte d'Azur, Nice, France; 3 Service de Néphrologie, Dialyse et Transplantation, Hôpital Pasteur 2, Centre Hospitalier Universitaire de Nice, Nice, France; 4 Department of Transplantation, Nephrology and Clinical Immunology, Edouard Herriot Hospital, Hospices Civils de Lyon, Lyon, France; 5 Institute of Molecular and Cellular Pharmacology, National Center for Scientific Research, University Côte d’Azur, UMR7275, Valbonne Sophia Antipolis, France; 6 Centre de Néphrologie et Transplantation Rénale, Aix Marseille Université, APHM, Hôpital Conception, Marseille, France; 7 Department of Nephrology, Dialysis and Transplantation, Montpellier University hospital, Montpellier, France; 8 University Center for Renal Diseases, Caen University Hospital, Caen, France; 9 Service de Néphrologie, Hémodialyse, Aphérèses et Transplantation Rénale, Grenoble Alpes University Hospital, La Tronche, France; 10 Nephrology Intensive Care, Department of Nephrology, Tenon Hospital, AP-HP, Paris, France; 11 Nephrology and Transplantation Department, Strasbourg University Hospital, Strasbourg, France; 12 Department of Nephrology and Transplantation, Bicetre Hospital, APHP, INSERM UMR 1186, Paris-Saclay University, Paris, France; 13 Nephrology, Dialysis and Transplantation Department, Gabriel Montpied Hospital, Clermont-Ferrand, France; 14 Department of Nephrology and Transplantation, Henri-Mondor Hospital, APHP, Créteil, France; 15 Department of Nephrology, Dialysis, and Renal Transplantation, Besançon University Hospital, Besançon, France; 16 Institut de Transplantation Urologie Néphrologie, Nantes University Hospital, Nantes, France; 17 Department of Nephrology, Transplantation, Dialysis et Apheresis, Bordeaux University Hospital, Bordeaux, France; 18 Department of Nephrology, Internal Medicine, Transplantation, Amiens University Hospital, Amiens, France; 19 Department of Nephrology, Brest University Hospital, UMR1227, Brest, France; 20 Department of Nephrology, Pontchaillou University Hospital, Rennes, France; 21 Nephrology, Dialysis and Renal Transplantation Department, Hôpital Nord, Saint-Etienne, France; 22 Department of Nephrology, Dialysis, and Organ Transplantation, CHU Rangueil, Toulouse University Hospital, Toulouse, France; 23 Department of Nephrology, Dialysis and Transplantation, University Hospital, Angers and Centre de Recherche en Cancérologie et Immunologie Nantes-Angers, INSERM, Nantes University, Angers University, Angers, France; 24 Regional Histocompatibility Laboratory, Saint Louis Hospital, AP-HP, Paris, France

**Keywords:** anti-PLA2R1 antibodies, kidney transplantation, membranous nephropathy, recurrence

## Abstract

**Introduction:**

Membranous nephropathy can lead to end-stage kidney disease, for which kidney transplantation is the preferred therapy. However, the disease often relapses, which can impact allograft survival.

**Methods:**

We conducted a prospective multicenter study in France involving 72 patients with membranous nephropathy who were awaiting and then underwent kidney transplantation. In addition, we established a retrospective validation cohort of 65 patients. The primary objective was to evaluate the prognostic significance of pretransplant anti phospholipase A2 receptor 1 (PLA2R1) antibodies on the recurrence of membranous nephropathy. The study also assessed the incidence rate, time to onset, and risk factors for recurrence, as well as allograft outcome.

**Results:**

The prospective cohort showed a 26% cumulative incidence of membranous nephropathy recurrence after a median follow-up of 23.5 months. This was confirmed by a 28% cumulative incidence after a median follow-up of 67 months in the retrospective cohort. A strong association was found between the presence of anti-PLA2R1 antibodies prior to transplantation and the risk of disease recurrence (risk ratio = 5.9; 95% confidence interval [CI]: 2.3–15.7; *P* < 0.0001). These results were confirmed in the retrospective cohort. Monitoring of anti-PLA2R1 antibodies in the immediate posttransplant period is of limited value, because recurrence occurred early in the first 6 months (median delay of 5 [3–14] months) after transplantation despite decreasing antibody levels.

**Conclusion:**

The presence of anti-PLA2R1 antibodies prior to transplantation was a strong predictor of recurrence of allograft membranous nephropathy. An individualized immunomonitoring and management strategy for kidney transplant candidates with anti-PLA2R1-associated membranous nephropathy should be considered.

## Graphical abstract

**Figure:**
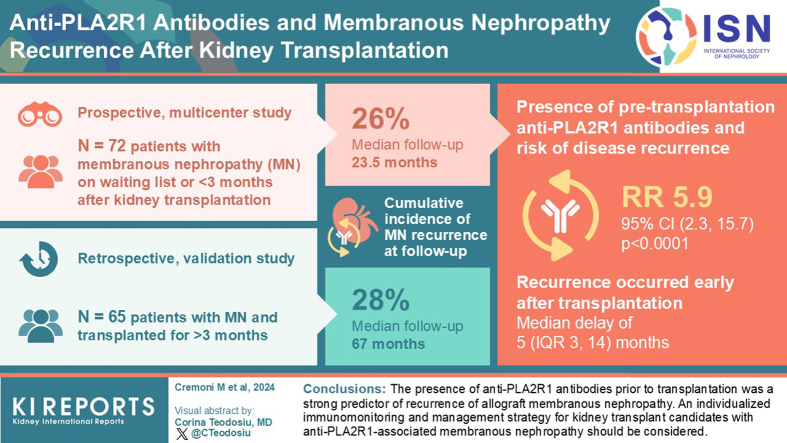

Membranous nephropathy is one of the most common causes of nephrotic syndrome in nondiabetic adults. This autoimmune kidney disease is characterized by subepithelial immune deposits containing IgG autoantibodies and complement fractions leading to alteration of the glomerular basement membrane structure.^1^ The autoantibodies target podocyte autoantigens, including the M-type PLA2R1 and thrombospondin type-1 domain-containing 7A in 70% and 3% of patients, respectively;^2^, ^3^, ^4^, ^5^ or other more recently discovered autoantigens.^6^ In the absence of treatment, one-third of patients progress to end-stage kidney disease,^7^ for whom kidney transplantation is the treatment of choice. Although allograft survival has improved in recent decades, recurrence or *de novo* glomerulopathy has become a major cause of allograft loss.^8^, ^9^, ^10^, ^11^ Recurrence of membranous nephropathy is common and can range from 30% to 50%, with higher rates reported by centers performing protocol biopsies.^12^, ^13^, ^14^, ^15^ Two peaks of posttransplant membranous nephropathy recurrence have been described: “early recurrence” within the first 6 months and “late recurrence” after 5 years.^14^^,^^16^ Early detection of patients likely to develop recurrence would be helpful in clinical practice. Risk factors for posttransplant recurrence have been suggested, such as older recipient age, steroid-free immunosuppressive regimen, low exposure to tacrolimus, living-donor transplantation, genetic factors such as PLA2R1 or HLA polymorphisms, and high levels of pretransplant anti-PLA2R1 antibodies.^14^^,^^15^^,^^17^, ^18^, ^19^, ^20^, ^21^, ^22^ Different anti-PLA2R1 antibody cut-off values have been proposed for identifying patients at risk: 45 RU/ml in the study by Quintana *et al.*^20^ based on 21 patients, and 29 RU/ml in the study by Gupta *et al.*^15^ which combined the previous cohort with 16 patients. However, others studies have not confirmed a strong correlation between anti-PLA2R1 titer and the risk of recurrence. Indeed, Debiec *et al.*^23^ showed that anti-PLA2R1 antibodies were implicated in only 5 to 10 recurrent patients. The retrospective nature of the studies, the small number of patients, the different techniques for detecting and quantifying anti-PLA2R1 antibodies, and the variable management, particularly for immunosuppressive treatments, may explain these differences. In addition, there is insufficient data on preventive treatment, including rituximab, for patients with membranous nephropathy and high levels of anti-PLA2R1 on the waiting list. Large-scale prospective studies are needed to clarify the usefulness of anti-PLA2R1 antibody monitoring before and after transplantation.

Here, we conducted a French multicenter study on posttransplant membranous nephropathy recurrence based on 2 cohorts: 1 prospective and the other 1 retrospective. The main objective was to assess the prognostic role of pretransplant anti-PLA2R1 antibodies for membranous nephropathy recurrence after kidney transplantation. We also evaluated the incidence rate, time to onset, clinical risk factors, and survival outcome of allograft in the case of recurrence of membranous nephropathy.

## Methods

### Study Design and Population

**Figure 1:**
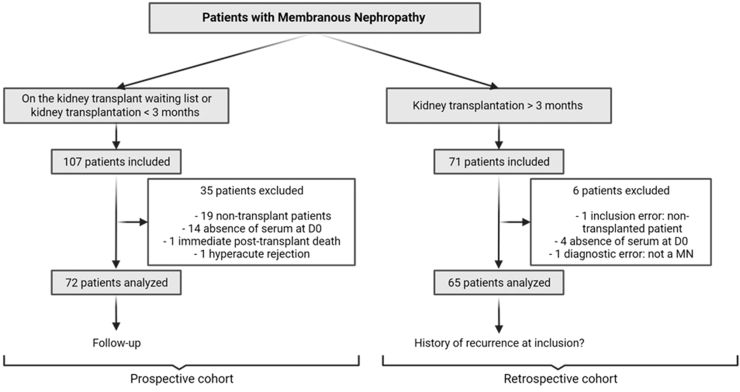
Flow chart showing patients enrollment. Patients with membranous nephropathy awaiting transplantation or transplanted for less than 3 months were included in the prospective cohort and followed for a maximum of 60 months. Patients with membranous nephropathy who had been transplanted for more than 3 months were included in the retrospective cohort. D0, day 0 of the transplantation; MN, membranous nephropathy.

Twenty-five French transplant centers (Amiens, Angers, Besançon, Bordeaux, Brest, Caen, Clermont-Ferrand, Grenoble, Lille, Lyon, Marseille, Montpellier, Nantes, Nice, Paris Henri Mondor, Paris Bicêtre, Paris La Pitié-Salpetrière, Paris Tenon, Rennes, Poitiers, Rouen, Saint-Etienne, Strasbourg, Toulouse, and Tours) participated in this prospective cohort study. Inclusion criteria were to have biopsy-proven membranous nephropathy, to be aged >18 years, to have signed an informed consent form, to be affiliated to a social security scheme, and to have 1 of the following 2 profiles: (i) patient awaiting kidney transplantation or transplanted for less than 3 months, or (ii) patient transplanted for more than 3 months, and in both cases to have a pretransplant serum available in the medical biology laboratory. Exclusion criteria were as follows: erroneous inclusion (i.e., the inclusion criteria were not met: no pretransplant serum available or patient not suffering from membranous nephropathy), no transplant during the follow-up period, and allograft lost within the first 7 days after transplantation. Patients included on the waiting list or within the first 3 months after transplantation were followed prospectively until the end of the study (maximum 60 months). Data from patients included more than 3 months after transplantation were collected retrospectively. Two cohorts were thus constituted: one prospective and the other retrospective (Figure 1).

The primary end point was to assess the prognostic value of pretransplantation anti-PLA2R1 antibodies for membranous nephropathy recurrence after kidney transplantation. The baseline time point was the day or the day before kidney transplantation, depending on the date of blood collection. To detect membranous nephropathy recurrence after kidney transplantation, monitoring visits were scheduled every 3 months during the first year after transplantation and then every 6 months until the end of the study. If a recurrence of posttransplant membranous nephropathy was clinically suspected, an allograft biopsy was required to confirm the diagnosis. Secondary end points included incidence rate; time to onset; and clinical risk factors for recurrence; as well as the management after recurrence, outcome, and allograft survival.

### Data Collection

Demographic, clinical, and biological data were collected at each transplant center by investigating physicians using paper case report forms, then transcribed by clinical research associates into secure, anonymized computer databases. We contacted the investigators at each center by e-mail or telephone to fill in any missing data. A pretransplant serum was obtained for all included patients, otherwise they were excluded from the study transplantation (i.e., serum from the day or 1 day before transplantation). Follow-up sera were routinely collected for patients followed-up prospectively. Sera were sent to the Nice University Hospital by the participating clinical departments or collected secondarily from laboratories.

### Laboratory Methods

Sera were tested for anti-PLA2R1 and anti thrombospondin type-1 domain-containing 7A autoantibodies by indirect immunofluorescence using the Euroimmun (Medizinische Labordiagnostika AG, Lübeck, Germany) kit. Quantification of total IgG anti-PLA2R1 antibodies was performed for all sera by enzyme-linked immunosorbent assay test, also developed by Euroimmun. It is important to note that at the time of the study (2011–2016), anti-PLA2R1 antibody testing was not routinely performed, and for most of the patients included, no antibody testing had been performed during follow-up. Autoantibody testing was performed centrally at Nice University Hospital after the study was completed. Thus, we did not know the cause of membranous nephropathy at inclusion and throughout follow-up, until the end of the study.

### Statistical Analyses

Qualitative variables were presented as numbers and percentages and compared using a Fisher exact test. Quantitative variables with a Gaussian distribution were presented as means and SDs and compared using the unpaired *t* test. Quantitative variables with a non-Gaussian distribution were presented as medians and interquartile ranges and compared with a nonparametric 2-tailed test (MannWhitney U). The Shapiro-Wilk normality test was used to determine if a variable had Gaussian distribution. A receiver operating characteristic (ROC) curve was used to define a threshold of anti-PLA2R1 antibodies at day 0, beyond which patients would be considered at risk of recurrence. Using the cut-off value determined by the ROC curve, a Kaplan-Meier analysis was used to estimate the relapse-free survival of patients with membranous nephropathy based on their anti-PLA2R1 antibody level. We used the Koopman asymptotic score to compute the CI of the relative risk. Statistical analyses were performed using GraphPad Prism 8.4.0 (GraphPad Software, Inc., San Diego, CA). All comparisons were 2-tailed, and the statistical differences were considered significant when *P*-value < 0.05.

### Study Approval

The study protocol conformed to the ethical guidelines of the 1975 Declaration of Helsinki and was approved by the appropriate institutional review committee (NCT01897961 2012-07). Written informed consent was obtained from participants prior to inclusion in the study. The clinical and research activities being reported are consistent with the Principles of the Declaration of Istanbul as outlined in the “Declaration of Istanbul on Organ Trafficking and Transplant Tourism.”

## Results

### Cohort Description

We included 178 patients with membranous nephropathy transplanted or requiring kidney transplantation from March 2011 to December 2016, in this French multicenter cohort study. One hundred seven patients (60%) were included while on the kidney transplant waiting list or within the first 3 months after transplantation and were followed-up prospectively for 23.5 (interquartile range [IQR]: 12–36) months. Seventy-one patients (40%) were included at least 3 months after kidney transplantation (67; IQR: 48–89 months posttransplant), and data collection was retrospective. Thus, 2 cohorts were constituted: 1 prospective and the other 1 retrospective. A total of 137 patients were analyzed after exclusion of respectively, 35 and 6 patients, that is, 72 patients in the prospective cohort and 65 patients in retrospective cohort. The flow chart depicting the recruitment of patients and their distribution in the 2 cohorts is provided in Figure 1. One hundred nine patients (80%) were male, and the mean age at transplant was 55 ± 13 years. Before kidney transplantation, 64 patients (47%) had received immunosuppressive therapy and 41 (30%) received antiproteinuric treatment. At transplantation, most patients received induction therapy with either lymphodepleting agent or anti-CD25 monoclonal antibody, and maintenance of triple immunosuppression including a calcineurin inhibitor. After transplantation, membranous nephropathy relapsed on the allograft of 37 patients (27%). Patients’ characteristics are detailed in Table 1. The 2 cohorts, prospective and retrospective, were analyzed independently.

**Table 1:** Characteristics of all study patients

Patient characteristics	Total patients (*N* = 137)
Basic characteristics	
Male gender (*n*)	109 (80%)
Age at transplant (yr)	55 ± 13
Time from diagnosis to ESKD (yr)	7 (3; 19)
Time on waiting list (mo)	14 (6; 35)
Previous transplantation (*n*)	16 (12%)
Immunosuppressive treatment before current transplantation (*n*)	64 (47%)
Corticosteroids	45 (33%)
Calcineurin inhibitors	25 (18%)
Rituximab	25 (18%)
Antimetabolites	16 (12%)
Chlorambucil	14 (10%)
Cyclophosphamide	6 (4%)
mTOR inhibitors	1 (0.7%)
Leflunomide	1 (0.7%)
Antiproteinuric treatment (*n*)^a^	41 (30%)
Characteristics at transplant	
Induction therapy *(n*)^b^	
Lymphodepleting agent (thymoglobulin, alemtuzumab)	66 (53%)
Nondepleting agent (anti-CD25)	56 (45%)
No induction	3 (2%)
Maintenance treatment *(n*)	
Calcineurin inhibitors therapy^c^	125 (98%)
Triple immunosuppression^c^	118 (92%)
Outcome	
Recurrences (*n*)	37 (27%)
Time of recurrence (mo)	13 (3; 53)
Allograft loss (*n*)	9 (7%)
Time from transplantation to allograft loss (mo)	31 (22; 84)

ESKD, end-stage kidney disease.

The number (and percentage) are indicated for categorical variables, mean (and SD) are shown for continuous variable with Gaussian distribution and median (and interquartile range) for continuous variables with non-Gaussian distribution.

a Antiproteinuric treatment: renin-angiotensin-aldosterone system blockers.

b 2 missing data.

c 9 missing data.

### Prognostic Value of anti-PLA2R1 Antibodies on Posttransplant Membranous Nephropathy Recurrence

#### In Prospective Cohort

**Figure 2:**
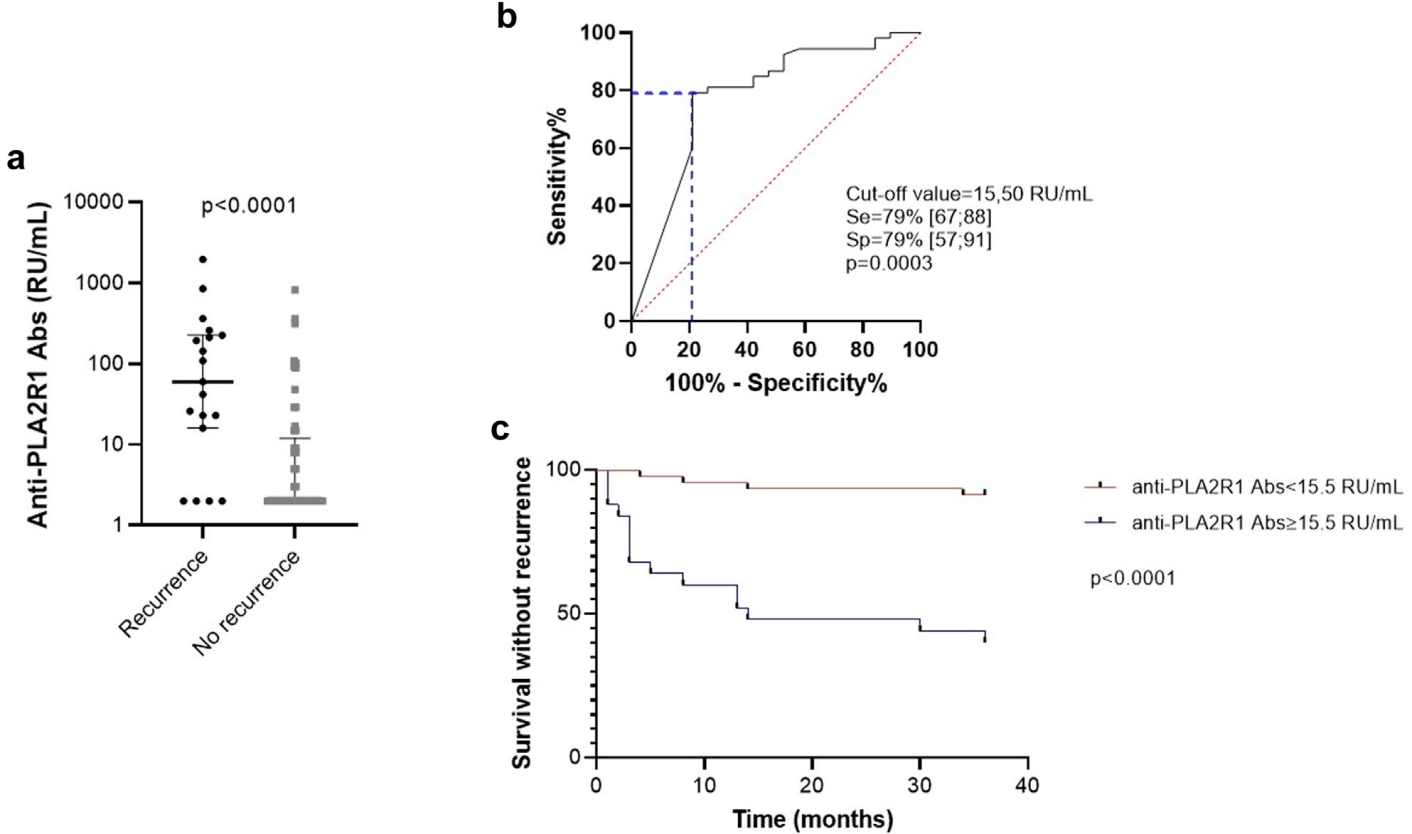
Prognostic value of baseline anti-PLA2R1 antibodies on membranous nephropathy recurrence after transplantation in the prospective cohort. (a) Anti-PLA2R1 antibody levels at baseline. Patients who relapsed after transplantation had significantly higher anti-PLA2R1 antibody levels on the day of transplantation than patients who did not relapse (60 [IQR: 16–226] RU/ml vs. 2 [IQR: 2–12] RU/ml; *P* < 0.0001). Statistical significance was determined by a Mann-Whitney test. (b) Receiver operator characteristic curve comparing baseline anti-PLA2R1 antibody levels between patients who will relapse and those who will not relapse. The anti-PLA2R1 antibody level cut-off value with the best prognostic performance at D0 was 15.50 RU/ml (sensitivity 79%, 95% CI: 67%–88%; specificity 79%, 95% CI: 57%–91%; area under the curve: 0.78; *P* = 0.0003). (c) Relapse-free survival of kidney transplant patients with membranous nephropathy. The cut-off value of 15.50 RU/ml, as determined by ROC curve, was used to distinguish patients at risk from those not at risk for relapse after transplantation (*P* < 0.0001). Kaplan-Meier analysis was used to estimate the relapse-free survival of patients with membranous nephropathy based on their anti-PLA2R1 antibody level. Abs, antibodies; AUC, area under the curve; CI, confidence interval; D0, day 0; IQR, interquartile range; PLA2R1, phospholipase A2 receptor 1; ROC curve, receiver operator characteristic curve; Se, sensitivity; Sp, specificity.

Nineteen of the 72 patients (26 %) experienced a recurrence of membranous nephropathy. Pretransplant anti-PLA2R1 antibodies were positive in indirect immunofluorescence in 28 of the 72 patients (39%). Recurrence occurred in 15 of 28 (54%) anti-PLA2R1-positive patients and in 4 of 44 (9%) anti-PLA2R1-negative patients (risk ratio = 5.9; 95% CI, 2.3–15.7; *P* < 0.0001). Patients with recurrent membranous nephropathy had significantly higher levels of anti-PLA2R1 antibodies at transplantation than those who did not relapse: 60 (IQR: 16–226) RU/ml versus 2 (IQR: 2–12) RU/ml, *P* < 0.0001 (Figure 2a, Table 2). Using a ROC curve, we demonstrated that the threshold value for the level of anti-PLA2R1 antibodies with the best prognostic performance at transplantation was 15.5 RU/ml (sensitivity 79% [95% CI: 67%–88%], specificity 79% [95% CI: 57%–91%], area under the curve: 0.78, *P* = 0.0003) (Figure 2b). We then used this cut-off value of 15.5 RU/ml pretransplantation to distinguish patients at risk from those not at risk for membranous nephropathy recurrence after transplantation (*P* < 0.0001) (Figure 2c).

**Table 2:** Characteristics of patients with membranous nephropathy, stratified by recurrence, in the prospective cohort (*n* = 72)

Characteristics	MN patients without recurrence (*n* = 53, 74%)	MN patients with recurrence (*n* = 19, 26%)	*P*
Demographic and clinical characteristics			
Age at transplant (yr)	55 ± 15	58 ± 10	0.06
Male gender (*n*)	42 (79%)	17 (89%)	>0.99
Time from diagnosis to ESKD (yr)	9 (3–20)	6 (1–12)	0.31
Time on waiting list (mo)	14 (7–36)	14 (6–37)	0.84
Time from transplantation to inclusion (d)	0 (−81 to 15)	0 (−214 to 2)	0.42
Induction therapy (*n*)^a^			
Lymphodepleting agent^b^	23 (43%)	8 (42%)	
Non-depleting agent^c^	20 (38%)	11 (58%)	0.37
No induction	3 (6%)	0	
Biological characteristics			
At baseline (before transplantation)			
Anti-PLA2R1 Abs by IFI (positive result)	12 (23%)	16 (76%)	<0.0001^d^
Anti-PLA2R1 Abs by ELISA (RU/ml)	2 (2–12)	60 (16–226)	<0.0001^d^
UPCR (g/g)	2.10 (0.98–5.69)	2.40 (1.36–5.76)	0.54
Albuminemia (g/l)	41 (35–46)	37 (36–40)	0.18
At mo 3 posttransplantation			
UPCR (g/g)	0.17 (0.11–0.34)	0.44 (0.15–1.95)	0.01^d^
Albuminemia (g/l)	43 (40–46)	40 (38–41)	0.002^d^
Serum creatinine (mg/dl)	1.64 (1.40–1.99)	1.74 (1.49–2.30)	0.24
GFR (ml/min per 1.73 m^2^)	46 (32–55)	40 (27–47)	0.07
At mo 6 posttransplantation			
UPCR (g/g)	0.16 (0.10–0.33)	0.47 (0.11–2.5)	0.04^d^
Albuminemia (g/l)	44 (40–45)	41 (38–44)	0.14
Serum creatinine (mg/dl)	1.66 (1.52–1.84)	1.73 (1.39–2.07)	0.93
GFR (ml/min per 1.73 m^2^)	43 (32–52)	38 (28–53)	0.79
At mo 12 posttransplantation			
UPCR (g/g)	0.22 (0.13–0.30)	0.53 (0.18–0.94)	0.04^d^
Albuminemia (g/l)	43 (42–46)	39 (37–41)	0.001^d^
Serum creatinine (mg/dl)	1.56 (1.44–1.81)	1.58 (1.40–2.23)	0.47
GFR (ml/min per 1.73 m^2^)	46 (40–58)	41 (28–58)	0.16
At mo 18 posttransplantation			
UPCR (g/g)	0.21 (0.08–0.30)	0.18 (0.14–1.01)	0.34
Albuminemia (g/l)	44 (42–46)	39 (37–41)	0.0005^d^
Serum creatinine (mg/dl)	1.57 (1.36–2.09)	1.69 (1.31–2.06)	0.70
GFR (ml/min per 1.73 m^2^, CKD-EPI)	46 (34–55)	40 (30–54)	0.47
At mo 24 posttransplantation			
UPCR (g/g)	0.16 (0.09–0.25)	0.41 (0.13–1.01)	0.14
Albuminemia (g/l)	45 (43–47)	39 (36–42)	0.0003^d^
Serum creatinine (mg/dl)	1.60 (1.36–1.84)	1.72 (1.38–2.24)	0.46
GFR (ml/min per 1.73 m^2^)	45 (39–62)	40 (28–55)	0.39
At mo 36 posttransplantation			
UPCR (g/g)	0.16 (0.10–0.34)	0.33 (0.11–0.61)	0.47
Albuminemia (g/l)	45 (42–48)	39 (36–44)	0.04^d^
Serum creatinine (μmol/l)	1.55 (1.32–1.66)	1.76 (1.20–2.43)	0.85
GFR (ml/min per 1.73 m^2^)	47 (34–59)	43 (26–66)	0.87
Outcome			
Time of recurrence (mo)	NA	5 (3–14)	NA
UPCR (g/g) at recurrence	NA	1.14 (0.54–2.39)	NA
Allograft loss within 36 mo follow-up, requiring hemodialysis (n)	1 (2%)	4 (21%)	0.02^d^
Time from transplantation to graft loss (mo)	33	24 (10–28)	NA

Abs, antibodies; CKD-EPI, Chronic Kidney Disease: Epidemiology Collaboration; ELISA, enzyme-linked immunosorbent assay; ESKD, end-stage kidney disease; GFR, glomerular filtration rate; IFI, indirect immunofluorescence; MN, membranous nephropathy; UPCR, urine protein-creatinine ratio.

The number (and percentage) are indicated for categorical variables, mean (and SD) are shown for continuous variable with Gaussian distribution and median (and interquartile range) for continuous variables with non-Gaussian distribution. Comparisons were performed using the unpaired 2-sided *t* test or Wilcoxon-Mann-Whitney U test according to data distribution for quantitative variables, and Fisher exact test for qualitative variables.

a 7 missing data.

b Thymoglobulin, alemtuzumab.

c Anti-CD25 monoclonal antibody (e.g., basiliximab).

d *P* < 0.05.

A closer look at the 19 patients who recurred showed that anti-PLA2R1 antibody levels declined rapidly after transplantation (Table 3). Despite this, recurrences occurred rapidly after kidney transplantation with a median delay of 5 [IQR: 3–14] months. Four patients were negative for anti-PLA2R1 and anti-thrombospondin type-1 domain-containing 7A antibodies at transplantation and at the time of recurrence, 4 patients had anti-PLA2R1 antibody levels > 15.5 RU/ml at transplantation and at the time of recurrence, and 11 patients had antibody levels > 15.5 RU/ml at transplantation but low (i.e., < 14 RU/ml), undetectable or unknown levels at the time of recurrence. Interestingly, 1 patient had high anti-PLA2R1 antibody levels at the time of transplantation (857 RU/ml) and relapsed within the first month while antibodies were decreasing (116 RU/ml the day of the kidney biopsy). His antibody level increased considerably (1338 RU/ml) at month 6, on an assay performed after transplantectomy.

**Table 3:** Posttransplant anti-PLA2R1 antibody levels in recurred patients from the prospective cohort

Recurred patients	Time from transplantation								
	D 0	Mo 1	Mo 3	Mo 6	Mo 12	Mo 18	Mo 24	Mo 30	Mo 36
1	109		15	5		7	6	9^a^	
2	364		52	11^a^	2	2	3		
3	23^a^		2		2	2	3		
4	2		2^a^		2				
5	1979		669	592	478^a^	246	478		
6	213		3^a^	2	2		2		
7	23		2	2	2	2	2		a
8	144		2^a^	2	2	2	4		
9	16	2	a	6	2	2	2	2	
10	261		2		2^a^	2	2	2	
11	2		2		2^a^				
12	194^a^	2	2	2	2		26		
13	226		8^a^	2	2	2	2		
14	60		5	8^a^	6	2			
15	42		a	2	2	2			
16	2			2^a^					
17	26		2	4	2^a^	2			
18	2		2	2	2	2	2	2	2^a^
19	857	116^a^	118	1338					

The values in boxes are anti-PLA2R1 antibody titers (RU/ml). Boxes are left blank when no serum was available for antibody testing.

a Antibody level closest to relapse.

As shown in Table 2, we compared characteristics of patients with and without recurrence, and highlighted that serum albumin levels in patients with relapse remained significantly lower than those in patients without recurrence, even at a distance from the recurrence, although within normal values. No other risk factors were identified in this cohort; and we did not observe any difference in induction or maintenance immunosuppressive treatments between patients with and without recurrence. The clinical and biological characteristics of patients who experienced a recurrence, before and after transplantation, are detailed in Supplementary Table S1.

#### In Retrospective Cohort

**Figure 3:**
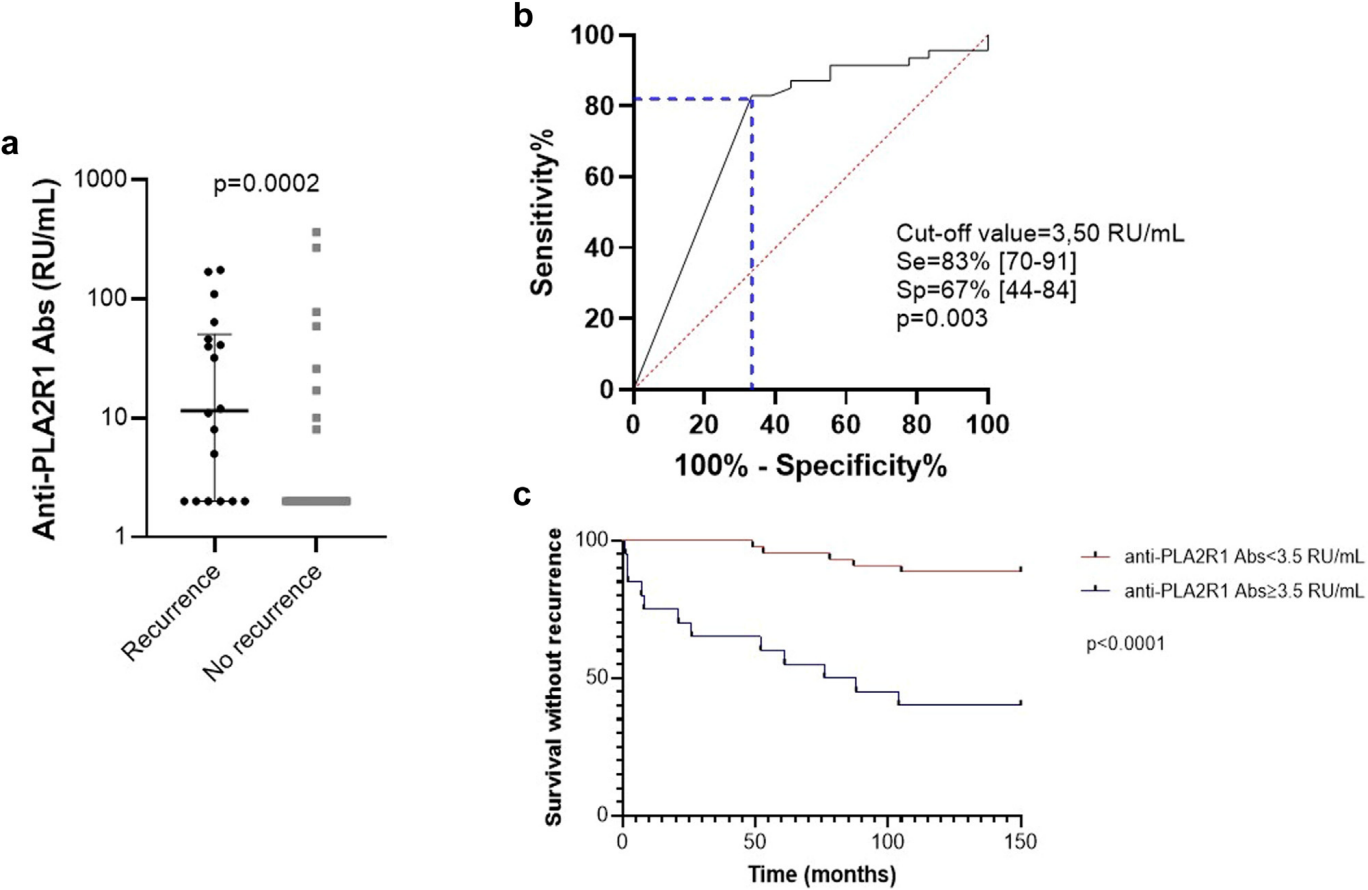
Prognostic value of baseline anti-PLA2R1 antibodies on membranous nephropathy recurrence after transplantation in the retrospective cohort. (a) Anti-PLA2R1 antibody levels at baseline. Patients who relapsed after transplantation had significantly higher anti-PLA2R1 antibody levels on the day of transplantation than patients who did not relapse (11.5 [IQR: 2.0–50.50] RU/ml vs. 2.0 [IQR: 2.0–2.0] RU/ml; *P* = 0.0002). Statistical significance was determined by a Mann-Whitney test. (b) Receiver operator characteristic curve comparing baseline anti-PLA2R1 antibody levels between patients who will relapse and those who will not relapse. The anti-PLA2R1 antibody level cut-off value with the best prognostic performance at D0 was 3.50 RU/ml (sensitivity 83% [95% CI: 70%–91%], specificity 67% [95% CI: 44%–84%], AUC: 0.74, *P* = 0.003). (c) Relapse-free survival of kidney transplant patients with membranous nephropathy. The cut-off value of 3.50 RU/ml, as determined by ROC curve, was used to distinguish patients at risk from those not at risk for relapse after transplantation (*P* < 0.0001). Kaplan-Meier analysis was used to estimate the relapse-free survival of patients with membranous nephropathy based on their anti-PLA2R1 antibody level. Abs, antibodies; AUC, area under the curve; CI, confidence interval; D0, day 0; IQR, interquartile range; PLA2R1, phospholipase A2 receptor 1; ROC curve, receiver operator characteristic curve; Se, sensitivity; Sp, specificity.

Eighteen patients (28%) experienced a recurrence of membranous nephropathy. Pretransplant anti-PLA2R1 antibodies were positive in indirect immunofluorescence in 20 of the 65 patients (31%). Recurrence occurred in 12 of 20 (60%) anti-PLA2R1-positive patients and in 6 of 45 (13%) anti-PLA2R1-negative patients. Patients with recurrent membranous nephropathy had significantly higher levels of anti PLA2R1 antibodies in enzyme-linked immunosorbent assay at transplantation than those who did not relapse: 12 (IQR: 2–51) RU/ml versus 2 (IQR: 2–2) RU/ml, *P* = 0.0002 (Figure 3a and Table 4). Using a ROC curve, we demonstrated that the threshold value for the level of anti-PLA2R1 antibodies with the best prognostic performance at day 0 in this cohort was 3.5 RU/ml (sensitivity 83%, 95% CI: 70%–91%; specificity 67%, 95% CI: 44%–84%; area under the curve: 0.74; *P* = 0.003) (Figure 3b). We then used this cut-off value of 3.5 RU/ml to distinguish patients at risk from those not at risk for membranous nephropathy recurrence after transplantation (*P* < 0.0001) (Figure 3c). In this cohort, recurrences occurred later, at a median of 52 (IQR: 8–83) months after kidney transplantation.

**Table 4:** Characteristics of patients with membranous nephropathy, stratified by recurrence, in the retrospective cohort (*n* = 65)

Characteristics	MN patients without recurrence (*n* = 47, 72%)	MN patients with recurrence (*n* = 18, 28%)	*P*
Demographic and clinical characteristics at baseline			
Age at transplant (yr)	54 ± 11	54 ± 10	0.93
Male gender (*n*)	36 (77%)	16 (94%)	0.16
Time from diagnosis to ESKD (yr)	12 (5–22)	3 (0.5–15)	0.13
Time on waiting list (mo)	14 (10–34)	3 (1–28)	0.11
Induction therapy (% lymphodepleting agent^a^)	24 (56%)	11 (65%)	0.53
Biological characteristics at baseline (before transplantation)			
Anti-PLA2R1 antibodies by IFI (positive result)	12 (26%)	8 (44%)	0.0001^b^
Anti-PLA2R1 antibodies by ELISA (RU/ml)	2 (2–2)	12 (2–51)	0.0002^b^
Outcome			
Time of recurrence (mo)	NA	52 (8–83)	NA
UPCR (g/g) at recurrence	NA	2.53 (0.90–6.01)	NA
Allograft loss within 100 mo (*n*)	2 (4%)	2 (11%)	0.57

ELISA, enzyme-linked immunosorbent assay; ESKD, end-stage kidney disease; IFI, indirect immunofluorescence; MN, membranous nephropathy.

The number (and percentage) are indicated for categorical variables, mean (and SD) are shown for continuous variable with Gaussian distribution and median (and interquartile range) for continuous variables with non-Gaussian distribution. Comparisons were performed using the unpaired 2-sided *t* test or Wilcoxon-Mann-Whitney U test according to data distribution for quantitative variables, and Fisher exact test for qualitative variables.

a Thymoglobulin, alemtuzumab.

b *P* < 0.05.

Patients’ characteristics with and without recurrence of membranous nephropathy are compared in Table 4. Despite low (e.g., < 14 RU/ml) or undetectable anti-PLA2R1 antibody levels in enzyme-linked immunosorbent assay the day of transplantation, 31% of patients had anti-PLA2R1 antibodies detectable with indirect immunofluorescence. An immunofluorescence positive result for anti-PLA2R1 on the day of transplantation was associated with recurrence (*P* = 0.0001, Table 4). Of note, 1 patient was found to have anti thrombospondin type-1 domain-containing 7A antibodies and did not experience recurrence during follow-up (41 months posttransplant).

### Treatment, Allograft Outcome, and Evolution

We had information on the treatment of membranous nephropathy recurrence for 15 of the 19 patients of prospective cohort: 11 did not have any therapeutic modification, 1 received renin-angiotensin-aldosterone system (RAAS) blocker alone, 2 received RAAS blocker combined with an increase in maintenance immunosuppressive treatment (1 increase in calcineurin inhibitor, and 1 introduction of mycophenolate mofetil), and 1 received RAAS blocker combined with rituximab. Of the 11 who had no change in treatment, 7 had a recurrence that remained stably subclinical, 2 had persistent nonnephrotic proteinuria, and 2 lost their allograft and returned to dialysis. The patient who had received RAAS blocker alone had persistent nonnephrotic proteinuria. The 3 patients who received RAAS blocker combined with an immunosuppressive treatment achieved complete remission.

We also observed in the prospective cohort that patients with membranous nephropathy recurrence after kidney transplantation had a higher risk of allograft loss (21% vs. 2%, *P* = 0.017) (Table 2). The risk of allograft loss in patients with recurrence correlated with anti-PLA2R1 antibody levels on the day of transplantation (857, IQR: 194–1979 RU/ml vs. 85, 24–223 RU/ml, *P* = 0.046). In the retrospective cohort, we did not observe any difference in the rate of allograft loss in patients with membranous nephropathy recurrence versus those without recurrence (2 [11%] vs. 2 [4%], *P* = 0.57) (Table 4).

We then analyzed the evolution of the 10 patients in the prospective cohort who had very high levels of anti-PLA2R1 antibodies (i.e., ≥ 150 RU/ml) on the day of transplantation. Among them, 7 (70%) experienced recurrence of membranous nephropathy, including 3 cases of allograft loss, representing a 43% allograft loss rate following recurrence. One patient died 9 months after transplantation, and although the cause of death was not provided, we considered him to be free of recurrence.

## Discussion

Membranous nephropathy is a rare autoimmune kidney disease that can lead to end-stage kidney disease in up to 30% of cases.^7^ The best solution for kidney replacement is kidney transplantation. Unfortunately, membranous nephropathy frequently relapses on the allograft,^12^, ^13^, ^14^, ^15^ which can lead to its loss, although the deleterious impact of recurrence on allograft function is controversial.^24^, ^25^, ^26^ Here, we describe the results of a French multicenter study of one of the largest cohorts of transplant patients with membranous nephropathy. Seventy-two patients were followed-up prospectively, making this the first prospective cohort of transplant patients with membranous nephropathy. Findings were then confirmed in a retrospective validation cohort of 65 patients. The rate of membranous nephropathy recurrence in this study was 27%, in line with what has been previously described.^12^, ^13^, ^14^, ^15^^,^^27^ The median time to onset of recurrence varied according to the cohort studied: 5 months after transplantation in the prospective cohort and 4 years after transplantation in the retrospective cohort (i.e., patients included more than 3 months after transplantation). These 2 delays correspond to the 2 peaks of posttransplant membranous nephropathy recurrence described in the literature (i.e., “early recurrence” and “late recurrence”).^14^^,^^16^ The difference in recurrence times between the 2 cohorts is likely due to the variable inclusion times in relation to transplantation: the prospective cohort was included before or less than 3 months after transplantation, whereas the retrospective cohort was included at a distance from transplantation (median 67 [IQR: 48–89] months posttransplant).

The main message of this study is that the presence of anti-PLA2R1 antibodies prior to transplantation is predictive of recurrence, regardless of titer, because the threshold of 15.5 RU/ml obtained after ROC curve analysis of antibody levels in the prospective cohort is close to the community-accepted threshold of positivity for enzyme-linked immunosorbent assay. It is desirable for patients to have a zero titer of anti-PLA2R1 antibodies on the day of transplantation. After kidney transplantation, anti-PLA2R1 antibody levels declined rapidly, probably due to induction immunosuppressive therapy and/or “sink” hypothesis.^28^^,^^29^ Despite this, patients experienced recurrence rapidly after kidney transplantation in the prospective cohort (median delay of 5 months). The results indicate that monitoring anti-PLA2R1 antibodies immediately after kidney transplantation is insufficient. This reinforces the idea of managing kidney transplant candidate patients so that they no longer have detectable antibodies on the day of transplantation. The optimal management strategy remains to be defined and must be discussed on a case-by-case basis. Monitoring of posttransplant anti-PLA2R1 antibodies is probably useful later, at a distance from induction immunosuppression, to detect “late recurrences.” Thus, we recommend to monitor the anti-PLA2R1 antibody level of patients with membranous nephropathy on the waiting list and subsequently discuss appropriate management based on each patient’s comorbidities and risk factors: for example, (i) propose a treatment such as rituximab to bring the anti-PLA2R1 antibody level to zero, particularly for living-donor transplantations, (ii) provide induction therapy tailored to the risk of recurrence (but there is no study on the best induction approach to avoid recurrence), (iii) offer close monitoring by immunomonitoring of anti-PLA2R1 antibodies and surveillance biopsies after transplantation, and promptly treat any recurrence. Recently, Hullekes *et al.*^30^ published the results of an international multicenter study (Post-Transplant Glomerular Disease Consortium) in which they found a one-third recurrence rate of posttransplant membranous nephropathy. The presence of anti-PLA2R1 antibodies on pretransplant sera (regardless of titer) was the major risk factor for recurrence. However, this study was retrospective and the number of subjects for whom pretransplant sera were available was limited to 46. Thus, our study confirms their results with a larger number of subjects and prospective follow-up.

Interestingly, 1 patient of the prospective cohort had high anti-PLA2R1 antibody levels at day 0 of transplantation (857 RU/ml) and relapsed within the first month while antibodies were decreasing (116 RU/ml the day of the kidney biopsy). His antibody level increased considerably (1338 RU/ml) at month 6, on an assay performed after transplantectomy. According to the data provided by the center that included him, no therapeutic modification, upward or downward, was made during this patient's posttransplant follow-up. This observation suggests that autoantibodies are sequestered in the allograft despite the decline in circulating antibodies. Staining of the anti-PLA2R1 antigen on graft biopsies, even in the absence of circulating antibodies, would likely be useful.

Among the risk factors for posttransplant membranous nephropathy recurrence, only the high levels of anti-PLA2R1 antibodies, although the threshold is not defined, seems to be consensual. Other risk factors have been proposed but not confirmed in subsequent studies.^15^^,^^17^^,^^20^^,^^27^^,^^31^ Here, we found no clinical risk factor predictive of allograft recurrence. Of note, albumin levels were lower in patients who were going to relapse and had relapsed, while remaining within normal values.

The higher risk of allograft loss after membranous nephropathy recurrence is controversial.^11^^,^^13^^,^^26^ We found in the prospective cohort that patients with recurrence after kidney transplantation had a higher risk of allograft loss. This observation was not confirmed in the retrospective cohort, in which recurrences were late. These results may suggest that patients with “early recurrence” are at risk of graft failure, but not those with “late recurrence.” These results need to be confirmed in a dedicated study.

This study has several limitations. First, the predictive value of anti-PLA2R1 antibodies is of interest only for patients with anti-PLA2R1-associated membranous nephropathy. However, given that we did not know patients' PLA2R1 status at inclusion (antibodies not routinely tested at the time the study was set up) and until the end of follow-up (centralized antibody assay in sera at day 0), we were unable to analyze a cohort consisting solely of patients with anti-PLA2R1-associated membranous nephropathy. This limitation is particularly relevant in the retrospective cohort, where it is challenging to determine whether the risk of posttransplant recurrence is attributable to pretransplant anti-PLA2R1 antibody positivity or to the etiology of anti-PLA2R1-associated membranous nephropathy compared to other etiologies. Second, the inclusion period chosen for the prospective cohort (3 months posttransplant) is not optimal; it should have been decided at the time of transplantation or within the first few days. Third, one-third of patients were excluded from the prospective cohort, either because they were not transplanted during the follow-up period, or because we did not have pretransplant serum (a mandatory inclusion criterion). Fourth, we did not have complete clinical data, particularly on the other causes of allograft loss potentially associated with recurrence, such as the occurrence of humoral rejection, or on the causes of death, limiting the impact of our findings on the risk of graft loss with recurrence. Fifth, only a few patients were treated for membranous nephropathy recurrence, notably with rituximab, which does not allow us to study the relevance and efficacy of relapse treatments. Furthermore, the risk of graft loss is likely to be overestimated due to the limited use of rituximab at the time of the study. This limitation is a consequence of the fact that patients were recruited prior to 2016. Sixth, the follow-up period of the prospective cohort was too short (median follow-up of 23.5 months) to observe late recurrences. Finally, the main limitations were the absence of a consensus definition of recurrence and the lack of a protocol biopsy. These limitations likely underestimated the number of posttransplant recurrences and resulted in delayed diagnoses. In addition, this led to heterogeneous sampling, because the clinical assessment of recurrence may vary from center to center.

In conclusion, these multicenter studies, including the first prospective study on membranous nephropathy recurrence after kidney transplantation, not only confirmed the predictive value of pretransplant anti-PLA2R1 antibodies but also showed that the presence of pretransplant anti-PLA2R1 antibodies could predict the recurrence regardless of their level. In view of the occurrence of early recurrences despite the decline in circulating antibody levels after transplantation, we suggest monitoring the anti-PLA2R1 antibody levels of patients with membranous nephropathy on the waiting list and discussing appropriate management according to each patient’s comorbidities and risk factors prior to transplantation.

## Disclosure

GL is an inventor on the patent “Diagnostics for membranous nephropathy.” BSP and GL are inventors on the patent “Methods and kits for monitoring membranous nephropathy.” All the other authors declared no competing interests.

## Supplementary Material

Supplementary File (PDF) Table S1. Detailed characteristics of recurred patients in the prospective cohort.

## Footnotes

Supplementary File (PDF)
**Table S1**. Detailed characteristics of recurred patients in the prospective cohort.
