# Supplementary material for: Anti Phospholipase A2 Receptor 1 Antibodies and Membranous Nephropathy Recurrence After Kidney Transplantation
Source: Kidney Int Rep. 2024 Sep 23;9(12):3427–38. doi: 10.1016/j.ekir.2024.09.012 (PMC11652070; doi:10.1016/j.ekir.2024.09.012)
Supplement: Supplementary File (PDF) — Table S1. Detailed characteristics of recurred patients in the prospective cohort. [file mmc1.pdf]

SUPPLEMENTARY MATERIAL

Supplementary Table S1. Detailed characteristics of recurred patients in the prospective cohort

Patients	Age*	Sex	At baseline (the day of transplantation)			At MN recurrence					Last observation carried forward			
			Anti-PLA2R1 Abs by ELISA (RU/mL)	Alb. (g/L)	Immunosuppressive treatment	Time from Tx to recurrence (months)	Anti-PLA2R1 Abs by ELISA (RU/mL)	UPCR (g/g)	Serum creatinine (mg/dL)	GFR (mL/min/1.73m²)	Follow-up (months)	Serum creatinine (mg/dL)	GFR (mL/min/1.73m²)	Graft survival
1	58	M	109	36	Basiliximab, CS, tacro, MMF	30	9	0.65	9.94	76	42	9.04	86	Y
2	68	M	364	37	Basiliximab, CS, tacro, MMF	5	11	5.52	16.61	53	43	15.82	43	Y
3	54	M	23	37	Basiliximab, CS, tacro, MMF	0	23		109.04	NA	36	23.16		Y
4	68	M	2		Basiliximab, CS, tacro, MMF	4	2	1.19	19.89		36	23.73	29	Y
5	62	M	1979	38	Thymoglobulin, CS, tacro, mycophenolate acid	13	478	2.50	27.80	23	18		NA	N
6	42	M	213	33	Basiliximab, CS, tacro, MMF	3	3	0.46	16.61	46	36	11.75	68	Y
7	63	M	23	47	Thymoglobulin, CS, tacro, mycophenolate acid	38		3.00			60			Y
8	74	M	144		Thymoglobulin, CS, cyclo, azathioprine	3	2	0.15	22.71	27	38	19.44	34	Y
9	75	F	16	33	Thymoglobulin, CS, tacro, MMF	2		1.09	27.12	18	21	25.99	19	Y
10	46	M	261	36	Thymoglobulin, CS, tacro, MMF	13	2		15.48	48	36	11.98	64	Y
11	49	M	2		Basiliximab, CS, tacro, azathioprine	14	2	1.01	13.90	59	14	13.90	59	Y
12	62	M	194	45	Thymoglobulin, CS, tacro, MMF	0	194		60.23	NA	27		NA	N
13	47	F	226	38	Basiliximab, CS, cyclo, MMF	3	8	0.30	12.54	48	35	12.54	50	Y

14	41	M	60		Basiliximab, CS, tacro, MMF	8	8	0.52	20.45		26	21.13		Y
15	53	F	42	37	Basiliximab, CS, tacro	3		2.05	19.32	27	18	15.82	36	Y
16	58	M	2		Basiliximab, CS, cyclo, mycophenolate acid	8	2	2.66	28.25	23	28		NA	N
17	66	M	26		Thymoglobulin, CS, tacro, mycophenolate acid	14	2	0.63	23.95	27	46	26.21	24	Y
18	57	F	2	35	Thymoglobulin, CS, tacro, mycophenolate acid	34	2	1.36	25.88	20	47			Y
19	65	M	857	41	Basiliximab, CS, cyclo, MMF	1	116	1.91	22.94	29	6		NA	N

Abs, antibodies; CS, corticosteroids; cyclo, cyclosporine; ELISA, enzyme-linked immunosorbent assay; F, female; GFR, glomerular filtration rate; MMF, mycophenolate mofetil; M, male; MN, membranous nephropathy; N, no; NA, not applicable; tacro, tacrolimus; UPCR, urine protein-creatinine ratio; Tx, transplantation; Y, yes.

* Age at the time of kidney transplantation
